# Integrative Review of Exercise at Altitude during Pregnancy

**DOI:** 10.3390/ijerph18179272

**Published:** 2021-09-02

**Authors:** Beth G. McManis

**Affiliations:** School of Nursing, Northern Arizona University, Flagstaff, AZ 86011, USA; Beth.McManis@nau.edu

**Keywords:** pregnancy, exercise, physical activity, altitude

## Abstract

Many competitive and elite athletes continue to train throughout their pregnancies and many visit or live at altitude. The purpose of this integrative review is to synthesize, analyze and critique published studies regarding the safety of serious recreational or elite athletes exercising at altitude while pregnant. Seven databases were searched, and 157 documents were located, which were screened for appropriateness and reduced to seven articles that met the criteria. The studies were analyzed based on maternal and fetal outcomes. Current recommendations for exercising at altitude were based on sedentary individuals who frequently did not have the expected physiological responses based on research on pregnancy and altitude. It is unknown whether competitive and elite athletes would have similar responses to exercise at altitude. More research on exercise at altitude on individuals with a variety of fitness levels is needed.

## 1. Introduction

There is a dearth of research regarding low- and moderate-intensity exercise at altitude during pregnancy and research is practically non-existent regarding vigorous exercise and athletes. Much of the research is extrapolated from non-pregnant individuals exercising at altitude or based on responses at rest. Researchers have investigated acute exposure (short-term visitors), relative short-term exposure (short-term residents), and long-term exposure (long-term residents) of altitude on resting values in pregnant women. Acute exposure can be a few hours to a few days. Short-term exposure entails acclimatization and some adaptation to altitude which can range from 3 days to permanent residency. Long-term exposure refers to natives of the altitude who have ancestors residing in the area for multiple generations. In addition, research has been conducted on pregnant women at sea level, lower altitude (<2500 m) and high altitude (>2500 m). However, some researchers deem lower altitude to be at 1600 m, at which the percent saturation of hemoglobin (HGB) or oxygen saturation (SaO2) is reduced at rest and exercise [1]. At altitudes equal or greater than 2500 m, hypoxia-related complications begin to occur since SaO2 begins to decrease exponentially with decreases in arterial oxygen tension (partial pressure of oxygen) (PaO2) [2]. However, individuals with chronic conditions such as pulmonary or cardiovascular disease can have these complications at much lower altitudes [2].

The guidelines regarding physical activity at altitude during pregnancy are primarily based on research performed on small samples, sedentary individuals or extrapolated from resting physiological data obtained on pregnant women at moderate to high altitudes [3,4]. The American College of Obstetricians and Gynecologists (ACOG) indicated that pregnant individuals, who live at sea level, can safely exercise up to 6000 ft (1829 ft) and pregnant individuals who live at higher altitudes may be able to safely exercise above 6000 ft [3]. In the Canadian guidelines, it is noted that it appears to be safe for individuals, who are pregnant and lowlanders, to perform moderate-intensity exercise at altitudes of 1800 to 2500 m, if acclimatized and should avoid exercising above 2500 m [4]. Elite and competitive athletes who train close to 2 h a day for at least 5 days a week at a moderate to vigorous intensity are strongly encouraged to consult their providers regarding the safety of continuing. It was a noted in both the ACOG and Canadian guidelines that there is sparse literature regarding this type of exercise program and more information is needed about exercising at altitude. However, providers have limited evidence to advise their patients, especially if the athlete is altitude training. Many endurance athletes live and train at high altitude in places such as Boulder, Colorado (1624 m/5328 ft), Colorado Springs, Colorado (1926 m/6322 ft), Flagstaff, Arizona (2134 m/7001 ft), Mammoth Lakes, California (2401 m/7880 ft), Iten Kenya (2377 m/7800 ft), San Luis Potos, Mexico (1899 m/6233 ft), and St. Moritz, Switzerland (1822 m/5978 ft) [5,6,7,8]. Athletes, both recreational and elite, can find little guidance on how to continue training once they become pregnant.

Much of the research on the resting physiological responses to altitude has been performed at 2500 m and higher, where most elite athletes do not train, unless they are training for trekking up a mountain. Depending on the physiological parameter, the response to altitude changes in a linear fashion from 2000 to 5000 m, and then the change becomes exponential [9]. Some of the key parameters to be considered when assessing the safety of exercising at altitude during pregnancy include the delivery of arterial oxygen content (CaO2), SaO2, ventilation, HGB, oxygen consumption ($\dot{V}$O_2)_, blood gases, and uterine blood flow [1]. The types of activities, the level of altitude and risks for fetal and perinatal complications also need to be addressed.

The purpose of this exploratory, integrative review is to synthesize, analyze and critique published studies regarding the safety of serious recreational or elite athletes exercising at altitude while pregnant. It is important for athletes and health care providers to understand the evidence that the exercise guidelines are based upon so they can assess the risks associated with exercising at altitude during pregnancy (ACOG, 2020) and develop a training program that will be safe for their situation.

An integrative literature review was chosen because it allows for a greater breadth of research to be analyzed and plays an important role in evidence-based practice in nursing and midwifery. Systematic reviews, while important to evidence-based practice, tend to focus on experimental studies, specifically randomized clinical trials, which can be limiting in exploratory studies such as this exploratory review [10,11]. Systematic reviews are commonly used to determine if an intervention is effective. However, in nursing and midwifery, questions regarding care and the impact of illness and treatment are of primary interest, which are more likely to be addressed by descriptive, observational, and qualitative research [10]. Integrative literature reviews should include diverse methodologies including clinical writing, such as case studies, reviews, editorials, and letters [11,12]. In fact, for this topic, given the lack of safety evidence, it would be highly unlikely that an IRB committee would approve an experimental design, nor would some participants desire to be assigned to an exercise group at altitude. Therefore, to be able to adequately synthesize and critique the current literature and inform future research, an integrative literature review was deemed to be the best option of analysis.

## 2. Materials and Methods

### 2.1. Search and Eligibility Criteria

This exploratory, integrative review was conducted using the methodology described by Toronto and Remington [13]. The following databases were searched on 4 May 2021: Academic Search Complete, Cumulative Index Nursing and Allied Health Literature (CINAHL Plus), Cochrane Library, Conference Papers Index, Dissertations and Theses: Full Text (ProQuest), PubMed (Medline), and Web of Science. The key words included pregnant or pregnancy, altitude, exercise, or physical activity (pregnancy OR pregnant AND altitude AND exercise OR Physical activity). The inclusion criteria for the type of document included (1) English language, (2) published since 1980, (3) experimental, quasi-experimental or non-experimental design, (4) altitude was at or above 1000 m, (5) participants were pregnant, and (5) moderate or vigorous exercise was performed. Studies were excluded if they were not conducted on humans or the emphasis was on other health conditions or chronic diseases. Ancestry, or backward searches, were conducted using Web of Science and reviewing the reference lists of the located articles.

### 2.2. Study Selection

Integrative reviews are purposefully broadly focused, pp. 11–20 in [13]. Since there is limited research in this area, a wide time span was used to capture the studies that exercise guidelines are currently based upon. Due to ethical concerns regarding research on pregnancy, experimental designs are often inappropriate, so a variety of research designs, including case studies, were included in the review. During a pregnancy, a woman experiences many physiological changes. The effects of exercise and altitude on the woman and her fetus can change during the pregnancy so studies conducted at any time during the pregnancy were considered. The population of interest was serious recreational and elite athletes who typically perform moderate and vigorous exercise; however, much of the research in this area has been conducted on individuals who are sedentary or who exercise for fun and fitness. These studies were included in the review since they provide some insight into the effects of altitude on exercising, pregnant women. Primary research was the focus of the review.

Searches were imported into Zotero v.5.096.2 (Corporation for Digital Scholarship, Vienna, VA, USA) [14] as the searches of the databases were being performed. Duplicates and articles not written in English were manually removed. Titles of the articles and then abstracts were screened to determine if the articles addressed human research, pregnancy, altitude, and exercise. Articles that focused on chronic conditions or other health issues also were removed. Lastly, full-text reviews were conducted, and articles were removed if they did not meet the inclusion criteria.

### 2.3. Risk of Bias in Individual Studies

The best method of appraising study quality in an integrative review is up for debate, though there is a consensus that the studies should be evaluated for quality and bias [11], pp. 44–45 in [13]. When diverse types of empirical sources are included in an integrative review, it is reasonable to evaluate the quality of the research when a discrepant finding is reported, or different types of quality criteria instruments could be used to evaluate the diverse type of methodology used across the studies [11]. It also is important to address the quality of the study in a meaningful manner in the final report. The quality of the studies was appraised through the lens of Rapid Critical Appraisal Checklists [15] and Joanna Briggs Institute Critical Appraisal tools [16]. Specific evaluations of the quality of the studies can be found on Appendix B.

### 2.4. Publication Bias

Many research studies that are completed are never published, pp. 45–55 in [13]. In addition, researchers who find non-significant or negative results are less likely to submit for publication or have their research published, pp. 45–55 in [13]. Therefore, databases containing dissertations, theses and conference papers were searched to find unpublished research. However, an inherent bias that cannot be avoided is the lack of research conducted on females, especially during pregnancy [17]. In addition, researchers have a difficult time obtaining human subject approval to conduct research on individuals who are pregnant since they are considered a vulnerable population by Institutional Review Boards [18,19].

## 3. Results

### 3.1. Study Selection

**Figure 1:**
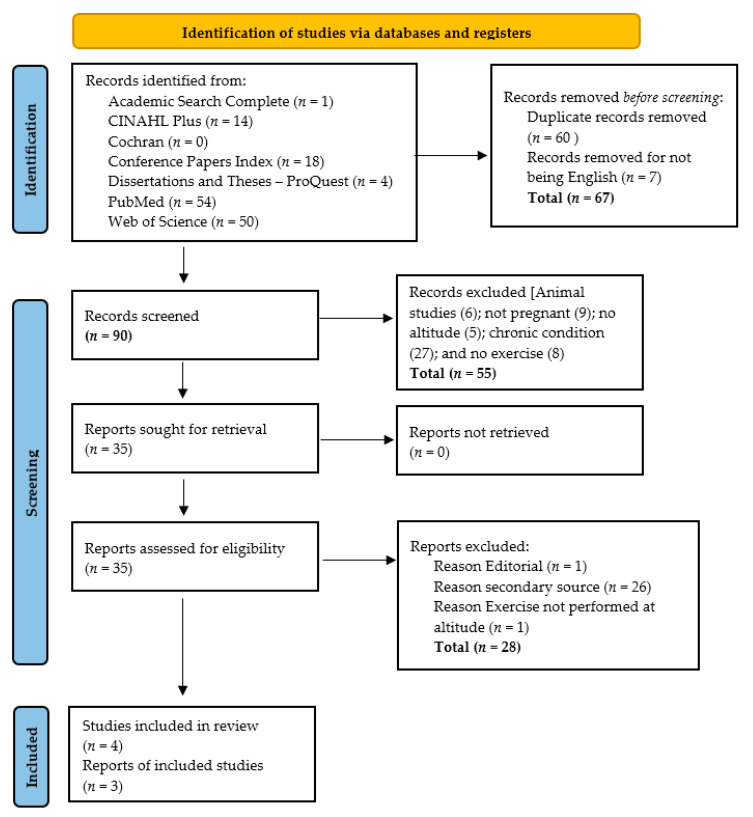
Search outcome and selection of articles for the integrative review. From: Page, M.J.; McKenzie, J.E.; Bossuyt, P.M.; Boutron, I.; Hoffmann, T.C.; Mulrow, C.D.; Shamseer, L.; Tetzlaff, J.M.; Akl, E.A.; Brennan, S.E.; et al. The PRISMA 2020 statement: An updated guideline for reporting systematic reviews. *BMJ* **2021**, *372*, n71, doi:10.1136/bmj.n71. ©Under the terms of Creative Commons Attribution License which permits unrestricted use and reproduction.

The selection of studies is illustrated in Figure 1. In the original search, 157 references were identified: one in Academic Search Complete, 14 in CINAHL Plus, zero in Cochran Library, 18 in Conference Papers Index, four in Dissertations and Theses, 54 in PubMed, and 50 in Web of Science. Sixty duplicates and seven references not in English were removed. The titles and abstracts were screened to determine if they met the inclusion criteria, and 55 references were removed. Full-text reviews were conducted on the remaining 35 articles. Twenty-eight secondary sources were removed. Of the remaining eligible seven references, three were articles that described studies that were done previously [9,20].

### 3.2. Description of Included Studies

The characteristics of the seven studies are provided in Appendix A. Three of the studies were conducted in the United States [20,21,22]. One study was conducted in Bolivia (step test) [23], one in Nepal (Sherpa) [24], and two in Switzerland (Swiss) [9]. All the studies were quantitative. Four were quasi-experimental with a repeated-measures design and incorporated exercise tests (maximal exercise study) [21] (Swiss) [9] and (Utah) [22]. One study was a survey [20], one was a correlational design which incorporated exercise testing [23] and one used a case study design [24]. In two of the studies, the prenatal values from exercise tests were compared to the postpartum values [22,24]. Exercise test values from sea level were compared to the altitude testing in three of the studies [21]. In one study, providers were surveyed [20].

The sample sizes for studies ranged from one participant [24] to 20 participants [23]. The studies with exercise tests had six to 20 participants, and three of the studies had only six to seven participants. Details about recruitment of the participants were not described in the quasi-experimental studies. The age of the participants was not reported in two of the studies [9]. All the studies involving exercise tests were conducted on participants who were in their third trimester. All the subjects were described as healthy and sedentary, although two studies described individuals who smoked throughout their pregnancies [9]. Lowlanders were recruited for the three studies [9,21]. Participants who lived at high altitude and were long-term residents, [23,24] and moderately low-altitude short-term residents [22] were used in three studies.

In five of the studies, exercise testing was conducted. A 6 min modified Harvard step test, in which the height of the step was reduced to compensate for the height of the participants and their late stage of pregnancy, was used in one study [23]. The step test was performed in LaPaz, which ranges from 3200 to 4000 m in altitude. The 3 min exercise tests in two studies were performed both at low altitude (1080 m/3500 ft) and moderate altitude (2228 m/7250 ft) on a bicycle ergometer in the Swiss Alps [9] within an hour of each other. The participants were transported from low altitude to moderate altitude via a 10 min cable car ride. The intensity was set at 25 and 50 W for the first and second study, respectively. Symptom-limited maximal exercise tests on bicycle ergometers at 25, 50 and 75 W for 5 min and then the workload was increased 25 W until volatile fatigue each at both sea level (54 m/180 ft) and altitude (1800 m/6000 ft) were incorporated in a study conducted in California [21]. The tests were conducted 2 to 3 days apart. In another study, participants completed two 6 min bicycle ergometer tests at 50 and 75 W and two 6 min walking treadmill tests at 2.5% and 12% grade at 1388 m (4553 ft) elevation in Utah during the same day and the pregnancy values were compared to the postpartum values [22].

In the case study, a 28-year-old Sherpa at 31 weeks gestational age, who resided in Nepal, was studied as she trekked from 3400 (11,154 ft) to 5300 m (17,388 ft) and back over 11 days [24]. Lastly, a study used a survey to assess the complications associated with physical activity at altitude during pregnancy [20]. Obstetrical care providers who provided care at altitude were surveyed [20].

### 3.3. Physical Activities, Work Loads and Aerobic Capacity at Altitude

The intensity of exercise and workloads varied greatly among the studies in which participants were required to exercise. The submaximal stepping test performed at 3200 m lasted 6 min and was equivalent to a mild to moderate level of intensity [23]. Some of the participants were unwilling or unable to maintain the required pace or complete the full test. The mean $\dot{V}$O_2_ was 12.0 mL·kg^−1^ min^−1^ and ranged from 7.9 to 15.6 mL·kg^−1^ min^−1^·. The Sherpa, who trekked from 3440 to 5300 m elevation and back over an 11 day period, performed an average of 270 min of moderate to vigorous physical activity per day at 31 weeks of pregnancy [24]. The trip was repeated 10 months later, and the Sherpa performed slightly less moderate to vigorous physical activity during the trek. In the maximal exercise test, an average of 98.86 and 82.14 W was performed at sea level and altitude (6000 ft/1800 m), respectively, which were significantly different (*p =* 0.03) [21]. These absolute workloads were described as low. The participants also cycled longer at sea level than at altitude. The mean peak $\dot{V}$O_2_ (mL· kg^−1^ min^−1^) at sea level (19.2 ± 1.46 SE) and altitude (16.82 ± 2.05 SE) were significantly different (*p =* 0.03) [21]. Oxygen consumption was not provided for four of the studies [9,22,24].

Obstetrical care providers from mountain communities and Denver, in a Delphi survey, recommended that individuals who are pregnant should use caution when performing activities that may lead to falls, especially skiing [20]. They noted that, for visitors who are pregnant, vigorous exercise at altitude is often associated with altitude-related complications, such as dehydration and falls. It was recommended that short-term visitors reduce or not exceed their usual level of physical activity and should wait 3 to 4 days to acclimate to the altitude before exercising. Some respondents advised that strenuous activity should only be attempted after acclimatizing for 2 to 6 weeks.

### 3.4. Cardiopulmonary Effects of Exercise at Altitude

#### 3.4.1. Cardiovascular Effects

The resting heart rates (HR) in the pregnant participants varied greatly. The Sherp’s resting HRs ranged from 69 to 77 beats/min during the 11 day trek and did not differ from the postpartum values [24]. These values do not reflect the typically higher HRs observed in pregnancy compared to postpartum [25,26]. However, decreased resting HRs are seen in pregnant individuals who perform aerobic exercise [26,27]. The mean resting HRs of the more sedentary participants were 93 beats/min (80 to 104 beats/min) [23], 93–95 beats/minute [21] and 101 beats/min [9]. When traveling from low altitude to moderate altitude, the resting HR did not change [9,21], which does not reflect previous findings [28].

In the three studies, in which exercise tests were performed at sea level or low altitude and moderate altitude, significant changes were not observed between the locations. An increase from a resting HR of 103 to 128 beats/min was observed at both low and moderate altitudes when the workload was 25 W in the submaximal test [9]. A similar trend was observed, but with slightly higher HRs, when the workload was increased to 50 W in the second study. In the maximal exercise test, the maximal HRs at sea level (M = 167.86, SE = 5.89) were slightly higher, but not significant, than the HRs at altitude (M = 161.64, SE = 7.03) [21]. The ending mean HR for the step test participants was 146 beats/min (SD = 9.0) [23].

Resting blood pressure was measured in only one study, which did not change from low altitude to moderate altitude [9]. Systolic blood pressure during exercise was 13 mmHg higher at moderate altitude (117 to 144 mmHg) than at low altitude (116 to 129 mmHg). A similar trend in the exercise diastolic pressures was observed. The increase in blood pressure in response to exercise is an expected finding [26].

Stroke volume (SV) and cardiac output (CO) values were reported in only one study [21]. The resting SV at altitude was significantly greater than at sea level (*p* = 0.04). A response to an acute exposure to altitude includes an increase in SV [28]. SV at 50 and 70 W did not differ significantly from the resting values. Smaller increases in SV were seen from resting to 25 W and resting to 50 W at altitude compared to sea level but were not significant. The CO was also significantly greater at altitude than at sea level (*p =* 0.04). CO also increases with an acute exposure to altitude [28]. A response similar to SV was found for CO regarding the exercise values.

#### 3.4.2. Hemoglobin Effects

Only the two studies conducted at high altitude reported HGB values. During the Sherpa’s trek, the HGB values ranged between 11.4 (4th day) and 14.0 g/dL (1st day) [24]. The mean HGB value for the step test participants was 14.4 g/dL (SD = 1.6 g/gL) [23]. These values represent hemodilution, which is typically observed in long-term residents of high altitude [29].

The Sherpa’s peripheral SaO_2_ was measured each day while resting. The values ranged from 83% to 94%, which were similar to the postpartum values that were obtained. The lowest value occurred after spending the night at 5160 m. During the postpartum trek, values were not measured above 4370 m [24].

#### 3.4.3. Pulmonary Effects

Respiratory rate (RR) was measured in all but one study [23]. The Sherpa’s resting RR ranged from 16 to 21 breaths over the 11 day trek, which was similar to the resting RRs found in two of the other studies [21,22]. The pregnancy and postpartum RRs for the Utah study were not significantly different. The RR typically does not change during pregnancy [30]. In the Swiss studies, the resting RR values were lower than that found in other studies (11 breaths/min–Study 1; exact value not reported for Study 2 [9]. The RRs did not differ between sea level/low altitude and moderate altitude [9,21]. In the first Swiss study, the RR increased from 11 to 20 breaths/min, but it was not reported if the difference was significant or not. The respiratory response was slightly higher in the second study but followed a similar pattern [9]. During the maximal exercise test, the mean RR increased from 21.50 breaths/min at rest to 38.50 breaths/min at maximal exercise when at altitude. The main effect for the RRs was significant with the exercise RRs higher than the resting rates (*p* = 0.001) for the Utah study, but the pregnancy status main effect for RR was not significant [22].

Tidal volume (TV) was reported in two studies [21,22]. The sea level TVs were not significantly different from the altitude values at rest, 25, 50 or 75 W [21]. In contrast, the exercise TVs were significantly greater at exercise than at rest for the Utah participants. In addition, the TV during pregnancy was significantly higher than the postpartum value (*p* = 0.001) [22]. TV typically increases by 40% during pregnancy [30] and is mostly responsible for increasing minute ventilation rather than the RR [31].

Minute ventilation was measured at the end of the step test and was 26.0 L/min (SD = 6.0). This was similar to the value found at altitude during the 25 W (M = 23.84 L/min, SE = 1.70) portion of the maximal exercise test. Minute ventilation did not differ between sea level and altitude at rest, 25 W, 50 W or the maximal level of exercise [21]. Minute ventilation at 75 W was significantly higher (M = 47.39 L/min, SE = 1.88) at altitude than at sea level (M = 42.57, SE = 1.52 L/min; *p* = 0.045) [21]. In contrast, minute ventilation across all participants was significantly less at rest compared to exercise in the Utah participants (*p* = 0.01) [22]. Additionally, the main effect for gestation status was significant, with the average pregnancy minute ventilation greater than the postpartum average (*p* = 0.001) [22]. The main effects for alveolar ventilation were also significant [22]. The pregnancy values across all rest and exercise conditions were significantly higher than postpartum (*p* = 0.001) and the exercise values were significantly greater than the rest values for all the subjects for alveolar ventilation (*p* = 0.01) [22]. The same was found for ventilatory equivalent ($\dot{V}$E/$\dot{V}$CO_2_) main effects. The pregnancy values were significantly greater than the postpartum values (*p* = 0.001) and the $\dot{V}$E/$\dot{V}$CO_2_ was higher during rest than during exercise (*p* = 0.01) [22]. The interaction effect was not significant for minute ventilation or alveolar ventilation. The main effects for dead space ventilation were not significant [22].

The studies being reviewed used various methods of measuring partial pressure of carbon dioxide (PCO_2_) and partial pressure of arterial carbon dioxide (PaCO_2_). Resting values of end tidal PCO_2_ during the 11 day trek ranged from 18 to 23 mmHg and were lower compared to 20 to 24 mmHg (3 measures at postpartum). These values represent hyperventilation, which is typically seen during pregnancy and at altitude. In the Swiss study, transcutaneous PCO_2_ was measured. It was unchanged from low altitude to moderate altitude and did not decrease during exercise. Hyperventilation did not appear to be present; however, actual values were not provided. The transcutaneous PO_2_ values decreased from 71 mmHg at low altitude to 58 mmHg at moderate altitude, which is an expected response to altitude exposure. There is a delayed response time of 45 to 60 s in the transcutaneous electrodes, especially for the PaCO_2_ electrode and if it is not heated appropriately [32]. It is possible that changes in PaCO_2_ were not detected at the end of the 3 min exercise test. PaCO_2_ was measured during the Utah tests and was significantly lower at rest during pregnancy (M = 26.5 Torr, SD = 2.2) than at postpartum (M = 35.3, SD = 2.4, *p* < 0.01). There was a significant interaction between gestational status and exercise (*p* = 0.001). Unlike at postpartum, the exercise values were relatively unchanged during pregnancy. The resting pH level was higher at rest at 37 weeks pregnancy compared to postpartum (*p* = 0.01). The main effect for exercise was significant with the pH decreasing from rest to exercise (*p* = 0.01). The bicarbonate values were significantly lower during pregnancy than postpartum at rest (*p* = 0.01). The significant interaction indicated that the decreases in bicarbonate from rest to exercise were smaller during pregnancy than postpartum (*p* = 0.001).

### 3.5. Perinatal and Fetal Effects

Fetal heart rates (FHRs) were reported before and after the exercise tests in three of the studies. The FHR at low altitude was 138 beats/min and increased to 142 beats/min at moderate altitude [9]. After the exercise sessions at 25 W, the FHR was 144 beats/min at both locations. At both workloads (25 and 50 W), two of the tracings were concerning. After the 25 W test, a 29-year-old participant, at 38 weeks gestation and a smoker, experienced a short, slight bradycardia episode and then decreased variability until a lower altitude was reached via the mountain cable car. After the 50 W test, a 33-year-old participant at 39 weeks gestation and a smoker had a 2 min bradycardia [9]. FHRs were measured before and after the maximal exercise tests at sea level and moderate altitude [21]. The FHRs did not differ before and after the tests or between the two altitudes. One participant experienced a 2 min bradycardia episode down to 105 beats/min after the test at moderate altitude, but the strip remained reactive the entire time [21]. No significant instances of fetal bradycardia were observed during or after the submaximal bicycle ergometer and treadmill exercise tests [22]. Specific FHR values for three of the studies were not reported [22,23,24].

Half of the participants (*n* = 6) reported feeling mild uterine contractions during the ascent to moderate altitude [21]. The contractions did not increase during or after the exercise session. After the maximal exercise test at moderate altitude, one participant, who was at 32 weeks gestation, experienced persistent and frequent uterine contractions and required observation in the hospital for 72 h and ultimately delivered at 39 weeks. It was reported in two of the studies that the participants had term deliveries and the infants had expected growth and development at the follow-up [21,24].

## 4. Discussion

In this exploratory, integrative review, only seven studies regarding exercise at altitude during pregnancy were located. Integrative reviews are typically exploratory in nature and this allows the inclusion of a variety of studies including non-experimental and quasi-experimental studies [11,13]. In this review, none of the included studies used an experimental design and, in fact, one was a case study, which is acceptable to use in an integrative review [12]. While the nature of the review was exploratory, care does need to be taken in interpreting and applying the results given the limitations of the studies.

The main limitations of the studies were that the sample sizes were small, ranging from six to 20 participants. However, in the Bellew study, 20 participants started exercise tests, but only 11 completed it [23]. In two of the studies, the method of selecting the participants was not described making it challenging to replicate the study and fully understanding the motivation of the participants during the exercise tests [9,21]. In addition, the researchers indicated that they selected sedentary individuals [9,21]. While many individuals are sedentary, it is unknown if these individuals would be apt to exercise at altitude and may not reflect individuals who seek out physical activity at altitude.

ACOG recommended in its guidelines that it appears safe for pregnant lowlanders to exercise up to 6000 ft (1829 m) and those who reside at altitude can most likely safely exercise above 6000 ft (1829 m) [3]. In the 2019 Canadian guidelines for physical activity, it was indicated that after appropriate acclimatization, it appears that moderate-intensity exercise during pregnancy does not adversely affect maternal and fetal well-being for altitudes up to 1800 to 2500 m [4]. Athletes, regardless of altitude, were advised that vigorous exercise up to 90% of maximal HR appears to be safe [3].

The ACOG and Canadian guidelines were based on recommendations from [21] Artal et al., which was reviewed in this article. In the Canadian guidelines, Jean and Moore [33] was also cited, who addressed questions and recommendations that clinicians may receive from their patients. Jean and Moore [33] cited Artal et al. [21] and two studies described in Huch [9], which were also reviewed in this article. Two different recommendations evolved from the two researchers. It was recommended that a pregnant individual should avoid visiting altitudes over 2500 m (8250 ft) during the first 4 to 5 days of a short-term visit. If the individual does desire to partake in physical activity upon arrival to altitude, it should be done at a lower altitude. However, the pregnant visitor was urged to be cautious and rest until acclimated [9]. Conversely, it was argued that performance in vigorous activities may decline, but it appears that brief episodes of moderate activity at altitude do not appear to be harmful to the fetus [21].

These recommendations were based on studies on sedentary pregnant individuals, who were short-term visitors to altitude, in their third trimester [9,21]. It was reported that the participants were healthy, even though at least two of them smoked throughout their pregnancies. The smoking status of the other participants was not reported. In two of the studies, the tests lasted only 3 min and required light to moderate exercise intensity, 25 and 50 W. Light exercise for women is between 21 and 48 W and between 49 and 76 W for moderate exercise [34]. The exercise that the women completed in the second part of the Swiss study was at the low end of moderate and only lasted 3 min, which would be equivalent to walking 3 km/h for a non-pregnant 70 kg person [34]. Data regarding oxygen consumption were not provided, so the actual fitness level of the participants could not be assessed. However, the length and intensity of the submaximal tests were well below a typical training session that a competitive or elite athlete most likely would do. In the other study, the workloads included 25, 50 and 75 W, and increased by 25 W until volatile fatigue was reached [21]. The amount of work completed at altitude by the participants was significantly lower than that completed at sea level [21].

In one of the studies, only sedentary participants were used because the sample would better reflect the large proportion of American women who are sedentary [21]. However, the exercise test may not have accurately reflected the effects of altitude on maternal and fetal health during exercise. The participants were required to perform the maximal test on a bike ergometer, which they were not familiar with. Secondly, the participants had to repeat the maximal exercise test at altitude 2–3 days after the sea level test and may have not reached maximal fatigue. This may have been because the women were not accustomed to exercising, especially on a bike ergometer, and may have been experiencing delayed-onset muscle soreness. It may have been beneficial to use a cross-over design with a wash-out period to minimize the possible effect of delayed-onset muscle soreness.

The HRs reached during the exercise tests were unexpectedly low. In the step test, the mean HR at the end of the test was 83% of the predicted maximal HR [23]. During the bicycle ergometer tests, the HRs were 86% (sea level) and 82.8% (altitude) of the predicted maximal HR and were not significantly different [21]. The participants’ maximal HRs were significantly lower than age-predicted maximal HRs for non-pregnant, but maximal HRs are slightly lower throughout pregnancy compared to nonpregnant HRs [35]. However, other researchers have also found that only 21% of the pregnant participants reached 95% of their age-related maximal HRs, which may be attributed to a pregnancy-related HR response [36]. While the research has been mixed on the HR response to high levels of exercise during pregnancy, the participants’ maximal HRs were lower than found in other studies. These responses may be attributable to the participant’s poor fitness levels. In addition, the workload at altitude at peak exercise was significantly lower than that at sea level indicating that some sort of compensation was occurring [21].

During the submaximal tests, the HRs ranged from 128 to 144 beats/min [9,23]. The 128 beats/min HR barely meets the low end of the recommended target HR range for healthy, pregnant individuals who have lower levels of fitness, but the target HR ranges are based on research performed near sea level [9,37]. Since HRs are typically lower at sea level than altitude [28], the submaximal test HRs appears to fall well below the recommended target HR range, indicating that the participants in these studies were poorly conditioned. Since it appears that the heart rate response during pregnancy and altitude may differ from the response in nonpregnant individuals at sea level, serious recreational and elite athletes should depend more on perceived exertion than HR when exercising during pregnancy [3].

In three of studies in which exercise tests were conducted, data regarding oxygen consumption were not measured [9,22], and therefore, the fitness levels of the participants could not be adequately assessed. During the step test, expired air was collected during the last 2 min and did not represent peak or maximal values [23]. The mean $\dot{V}$O_2_ for the participants was 12.0 (mL·kg^−1^ min^−1^). More data were obtained from the maximal exercise tests performed at sea level and altitude [21]. The $\dot{V}$O_2peak_ measured in liters per minute, was significantly lower at altitude than at sea level. The oxygen consumption has been found to decrease 3% for every 1000 ft above 5000 ft [21]. The 13% difference was much greater than what has been observed in non-pregnant women at the same altitude, which may have been caused by additional metabolic demands associated with pregnancy and the subjects’ poor fitness levels [21]. The mean $\dot{V}$O_2 peak_ values were 19.21 and 16.82 mL·kg^−1^ min^−1^ at sea level and altitude, respectfully. These values are below the 25th percentile, 21.0 mL·kg^−1^ min^−1^ (ages 20–29 years) and 19.6 mL· kg^−1^ min^−1^ (ages 30–39 years), which are associated with unfit pregnant individuals [37]. They also are much lower than that found for well- trained pregnant athletes who had $\dot{V}$O_2max_ (mL·kg^−1^ min^−1^) values ranging from 27.4 to 52.6 [38]. However, these comparison values are based on research conducted on individuals who were 15 to 22 weeks gestational age and were tested at sea level [37,38].

Healthy, physically active individuals who are pregnant frequently do not reach the primary or secondary endpoint criteria for a maximal exercise test due to emotional and physical barriers even though the participants felt they reached their maximal effort [36]. The criteria for the end points of maximal tests may need to be changed or other possible physical or emotional barriers affecting performance may be affecting performance [36]. This may be especially true for late-term sedentary, pregnant individuals who are not accustomed to exercising. This study was conducted in 1994 when many women were discouraged to exceed 140 beats/min when exercising and this may have created a psychological barrier for the participants to reach maximal effort. It is challenging to extrapolate these data to individuals who are fit and desire to exercise at longer and higher workloads. Maximal and submaximal exercise tests at altitude using sedentary and conditioned pregnant individuals across all gestational ages needs to be conducted to help determine the safety of exercising at altitude. Precautions must be taken in the maximal tests since fetal bradycardia, a high umbilical artery pulsatility, and a 50% reduction in mean uterine artery volume blood flow occurred when elite, pregnant athletes exceeded 90% of the maximal maternal heart rate in a submaximal exercise test conducted at sea level [39]. However, given the study involved only six athletes, more research regarding the safety of exercising beyond 90% of the maximal heart rate is also needed.

Resting SV and CO were significantly higher at moderate altitude than at sea level but did not change significantly during exercise [21]. CO increases in response to an acute exposure to altitude in non-pregnant individuals [28]. However, at high altitude (4370 m), resting CO and SV increased during pregnancy and peaked at 25 weeks but were lower at altitude than at sea level [40]. An increase in resting CO until the third trimester at sea level has been demonstrated [25]. Resting SV usually increases until the second trimester in lowlanders and augments CO during exercise [26]. It is possible that the response at rest during pregnancy at high altitude differs from the response at moderate altitude. The participants did not have nonpregnant controls, so it is not possible to determine if the CO and SV were increased because of pregnancy. Compared to nonpregnant and postpartum individuals, pregnant individuals had similar CO and SV responses to low and moderate submaximal exercise at sea level [41] but this was not observed in the reviewed study. It appears that the participants either had low reserve for these parameters or blunting occurred. More research on the cardiac responses during pregnancy, especially at altitude in well-trained individuals, is needed.

For several other physiological parameters, the results differed from what was expected. Surprisingly, only some of the women experienced the typical pregnancy and acute altitude exposure response of hyperventilation at rest. Pregnancy-induced hyperventilation, driven by estradiol and progesterone, occurs fairly early in pregnancy. Ventilation, which can increase by as much as 50% at sea level is due to a 40% increase in TV, and results in an increased Ve/VCO_2_ slope [30,42]. At sea level, an increase in PaO_2_, a decrease in PaCO_2_, and a relatively unchanged SaO_2_ results in pregnancy-induced hyperventilation [1,2]. Plasma bicarbonate and hydrogen ion concentration is decreased and pH is unchanged or slightly elevated, resulting in mild respiratory alkalosis during pregnancy [30,42], but it is reported elsewhere that renal bicarbonate excretion is increased [43] It appears that there is a complex interaction involving alterations in the acid–base balance and increases in non-chemoreflex and central chemoreflex drives to breathe that results in pregnancy-induced respiratory alkalosis instead of normal respiration or respiratory acidosis [42].

Beginning at approximately 1600 m, PaO2 increases and PCO_2_ and SaO_2_ decreases relative to sea level but is higher than that found in non-pregnant women at sea level [1]. The increase in SaO_2_ relative to non-pregnancy helps preserve the oxygen content like that found in nonpregnant women at altitude [1,2]. However, the PaO_2_ and SaO_2_ observed during pregnancy are lower at altitude than that found at sea level during pregnancy [1].

In the Utah study, minute ventilation was higher in pregnancy than postpartum and the exercise values were higher than the resting values [22], as expected. However, in the maximal exercise study, the minute ventilation values were significantly higher only at 75 W, and the values at sea level and altitude did not differ [21]. The resting TV also did not differ from the exercise values. It appears that the participants did not have hyperventilation normally observed in pregnancy and with an acute exposure to altitude. This finding has not been reported by others [22,30,42].

The resting RR at altitude is the same during pregnancy and the non-pregnant state [30,44] and does not start to significantly increase until above 3500 m [45]. At 36 weeks gestation, the normal range for resting RR is between 6 (3rd percentile) and 24 (97th percentile) with 16 at the 50th percentile [46]. During the 25 W test, the Swiss participants’ RR was within the normal range (the rate during the 50 W test was not reported by Huch, 1996). This level of exertion is far below the level of intensity of exercise a competitive recreational or elite athlete might perform. The RRs during the maximal test and Utah studies ranged from 28 to 41 breaths/min. Unexpectedly, the resting RR values for the pregnant and postpartum participants were significantly different, which were not explained.

The PCO_2_ responses in the reviewed studies did not reflect what has been observed elsewhere, except in the Sherpa, who displayed hyperventilation throughout the trek as indicated by the end tidal CO_2_ values. In the Swiss studies, the PO_2_ values decreased from low altitude to high altitude as expected; however, the PCO_2_ did not decrease at altitude or with exercise indicating that the participants did not have the expected hyperventilation. It is possible that the transcutaneous electrodes did not adequately detect changes in PCO_2_ during the short exercise session. The resting PaCO_2_ values were significantly lower during pregnancy than postpartum; however, the exercise values were unchanged from rest during pregnancy but significantly changed during postpartum [22]. The sedentary participants did not exhibit hyperventilation as expected, but it was observed in the trained Sherpa, who also was at a much higher altitude than the other participants. It seems that altitude would have a greater impact on a sedentary individual, than a trained athlete, though the greater altitude may have a greater impact on PCO_2_. The short-term residents who participated in the Utah study that took place at the lowest altitude, may have acclimated to the altitude and the exercise load may not have been sufficient to elicit an effect. In the Swiss and maximal exercise studies, compensation may have been taking place in the poorly conditioned participants, the equipment may not have detected the changes or the change in altitude may not been large enough to elicit a response. The effects of PO_2_ and PCO_2_ are especially important since they are indicators of hypoxia, which may adversely affect the fetus. Athletes who frequently exercise at a high intensity may be at risk for reduced blood flow to the fetus, especially at altitude. Therefore, it is important to know if they experience the same responses as found in these studies to help determine if it is safe to train at higher intensities.

FHR was measured in three of the studies before and after the exercise sessions. The resting FHRs were relatively unchanged from sea level to moderate altitude and did not change during the exercise tests. However, one participant in each of the three studies had a fetal bradycardia. Two of the episodes lasted 2 min and the third was a short bradycardia followed by a non-reactive strip for a short period. All episodes occurred at altitude. All episodes were in sedentary individuals and two of the participants were smokers. There is limited research on fetal response to exercise. In a study of six elite athletes, two women experienced decelerations after exceeding 90% of their maximal HR and the FHR returned to normal within 10 min. There were no further sequalae [39]. The significance of this finding is unknown [47], but is the basis of the ACOG recommendation that athletes should avoid exceeding 90% of maximal HR during exercise [3,39]. In a systematic review of healthy pregnant individuals, exercise was found to have a positive or neutral effect on FHR [48]. There was an increase in FHR immediately following an acute bout of exercise in five of the 33 studies and in three studies it was reported that the sedentary individuals were more likely to have an increase in the FHR after exercise. No statistically significant differences in the number of decelerations were reported in any of the studies [49]. Although the research on the fetal response to exercise is limited, a few studies, with very small sample sizes and reports of bradycardia and decelerations, seem to largely influence the ACOG and Canadian recommendations. While only 7.8% to 11% of pregnant individuals perform regular, moderate-intensity exercise during pregnancy [43], research regarding the impact on fetal well-being needs to be conducted on individuals with a variety of fitness levels.

The presence of uterine contractions was reported in two of the studies [9,21]. Half of the participants experienced mild uterine contractions during the ascent to altitude in the cable car, but the frequency did not increase during or after exercise [9]. After the maximal exercise test at altitude, one participant had frequent, persistent uterine contractions that subsequently required hospitalization [21]. A complication frequently seen by providers located in mountainous communities is dehydration, which is frequently associated with an onset of contractions [20]. The six participants in the Swiss study may have been experiencing contractions because of mild dehydration or because of the rapid ascent to altitude. They had taken a 130 km (80 mile) car ride to get to the cable car and may have restricted fluids for and during the car ride, resulting in possible dehydration. In addition, since most individuals do not usually travel by cable car to altitude, this may not be a significant finding. Uterine contractions have been found to be more related to the type of exercise than the intensity of exercise [50]. Uterine contractions were more likely to occur after riding a bicycle ergometer or running, which were the forms of exercise performed during the maximal exercise test [21,50]. However, in a Danish study, it was found that all types of exercise, except horseback riding, were associated with a reduced risk of preterm birth. There was a reduced risk of preterm birth associated with those who exercised, and the amount of exercise was not associated with the risk of preterm birth [51].

## 5. Conclusions

The current ACOG and Canadian guidelines regarding exercising at altitude while pregnant, suggest that short-term visitors may safely exercise up to 6000 ft (1800 m) and short-term residents may exercise above 6000 ft or that short-term visitors may perform moderate exercise up to 1800 to 2500 m (6000–8250 ft) after acclimating. It is also suggested that regardless of the location, pregnant individuals should avoid exceeding 90% of their maximal HR. These recommendations are primarily based on two studies that were conducted at 1800 and 2228 m with sedentary pregnant individuals. The guidelines are written primarily, and understandably, for most pregnant individuals, who are sedentary. One study appeared to use slightly more fit individuals, but it was performed at 1388 m on short-term residents. The only research concerning exercise at altitude in moderately fit or elite pregnant individuals that could be found was on a Sherpa who completed a trekking trip at 3400 to 5300 m.

In the reviewed research, the participants were poorly conditioned and the workloads for many of the tests were low. Hyperventilation was not observed in many of the participants, which is usually observed in pregnancy and altitude. The Sherpa, who was an elite endurance athlete, did have hyperventilation, but the trek was performed at a much higher altitude. The sedentary participants did not have many of the expected physiological responses to exercise such as increased stroke volume, cardiac output and decreased PCO_2_. It is unknown if these unexpected responses were due to the combination of altitude and pregnancy, poor conditioning or some type of compensation occurring. Given these exploratory results, it is difficult to develop recommendations for exercising at altitude for those competitive recreational and elite pregnant athletes and even sedentary and active individuals who are pregnant. Complicating the development of recommendations and guidelines, as illustrated in this review, is that it appears pregnant individuals respond differently to exercise at altitude depending on factors such as age, fitness level, health status, altitude level, type of exercise, duration, intensity, and training response to altitude. Care must be taken in making recommendations regarding exercising at altitude during pregnancy since there is so little supporting evidence and research.

Since many athletes and non-athletes venture to high altitude for short-term visits and extended training periods, more research is needed regarding the maternal and fetal responses to moderate and vigorous exercise at altitude. Individuals who are pregnant are considered a vulnerable population by the IR. Exercise has been thought to be a threat to fertility and pregnancy so many researchers have avoided conducting research in this area, especially regarding exercising at altitude. More research is need regarding exercising at altitude throughout pregnancy in sedentary, active, serious recreational and elite athletes. This research needs to be on short-term visitors, short-term residents and long-term residents who participate in a variety of activities at various altitude. Because so little is known about exercising at altitude during pregnancy, the initial studies most likely will need to be observational or retrospective in nature.

## Data Availability

No data was created in this study. Data sharing is not applicable to this article.

## Appendix A

**Table A1:** Summary of studies regarding the effects of exercise at altitude during pregnancy (Part 1).

First Author, Year, Country	Purpose	Methods	Study Sample
Artal 1995 California, United States	Determine if the cardiopulmonary responses, aerobic work capacity, work efficiency and FHR response while pregnant differ between altitude (6000 ft/1829 m) and sea level.	Quantitative study. Quasi experimental, repeated-measures design.	Seven pregnant, lowlander, females in third trimester, 5 Hispanic and 2 White, sedentary, completed the study. Two subjects dropped out due to personal reasons after the first test and the third had persistent uterine activity after the first test. The mean age was 24.86 ± 2.18 SE and gestational age was 33.86 ± 1.46 SE.
Ballew 1984 La Paz, Bolivia	One of the purposes of this dissertation was to investigate the relationship between maternal exercise performance at high altitude and body composition of the newborn.	Quantitative study. Non-experimental, correlational design.	Twenty pregnant, females of Indian ancestry, born and living in La Paz, Bolivia (3000 m or more), in the seventh or eighth month of pregnancy, 18 to 35 years of age, participated in the exercise portion of the study. Potential subjects were excluded for infections, hypertension, anemia, and respiratory diseases.
Davenport, 2018 Everest Base Camp, Nepal	Assess the physical activity, sleep and cardiovascular function in a female, native, trekking Sherpa guide during a trek from 3400 to 5300 min Nepal Himalaya.	Quantitative study. Case study.	A 28-year-old primiparous female at ~31 weeks who lived most of her life above 3450 m, with grandparents and mother who were also born and lived at high altitude. The participant was the only female Sherpa who trekked and guided tourists from the Everest Base Camp. She was also an elite competitive endurance athlete at the time.
Huch, 1996 (Baumann 1985 and Bung, 1987 as reported by Huch) Swiss Mountains and lab, Switzerland	Describes two studies that investigated the effects of exercise in high altitude on sedentary pregnant individuals. The first study took place during a short-term trip to altitude and the second study was in a chamber that simulated moderately high altitude.	Quantitative study. Quasi-experimental, repeated-measures design.	First study: Twelve sedentary pregnant lowlander females with uncomplicated pregnancies. Ten were primipara and two were multipara. Gestational ages ranged from 30 to 39, with a mean of 36 weeks. Mean age of participants was not reported. Second study: Six pregnant females with gestational ages ranging from 31 to 38 weeks. Four were primipara and two were multipara. Second Study: Due to the bradycardia in the first study, the second study was conducted in a in low pressure chamber. Four primiparas and two multiparas between 31- and 38-weeks gestational age participated. Mean age of participants was not reported.
Niermeyer 1999 Colorado, United States	Determine the observed pregnancy complications and risk factors for pregnant individuals who visit high altitude.	Quantitative study. Survey design.	Respondents included 12 Colorado obstetrical care providers who worked in mountain communities and Denver referral lefts.
Pivarnik 1992 Utah, United States	Determine the alterations in maternal respiration and blood gases in pregnant individuals while performing aerobic exercise at moderate altitude during the late third trimester and postpartum.	Quantitative study. Quasi-experimental, repeated-measures design.	Seven, healthy, primigravid, untrained individuals at 36.9 ± 0.9 weeks of gestation. Average age was 21.0 ± 1.9 years. All participants lived at 1388 m elevation and fully acclimatized.

Note. FHR = fetal heart rate.

**Table A2:** Summary of studies regarding the effects of exercise at altitude during pregnancy (Part 2).

*First Author, Year and Country*	Data Collection/Type of Intervention	Key Findings
Artal 1995 California United States	Symptom-limited maximal exercise tests on a bicycle ergometer at sea level (180 feet) and at 6000 feet 2 to 4 days later. Brief medical exam, including ECG, BP assessment and 30 min of fetal monitoring prior to exercise test and CO assessments. Exercise protocol: Rest on bicycle ergometer 5 min. Pedal 50 revolutions/min for 5 min at 25, 50, and 75 W until volitional fatigue. Peak $\dot{V}$O_2_, oxygen consumption, ventilation, HR, CO, SV, TV, and RR measured at rest, at each work level, maximal exercise, and recovery. BP, uterine activity, and FHR measured before and after each test.	Sedentary, lowlander women performed less work (p = 0.03), had a lower peak $\dot{V}$O_2_, (L/min: p = 0.03; mL/kg/min: p = 0.04), and $\dot{V}$CO_2_max (p = 0.03) at altitude compared to sea level. The oxygen consumption was significantly lower at altitude than sea level but was similar at rest and each workload. There were no significant differences between sea level and altitude for HR, TV, RR, and plasma lactate. Ventilation was significantly higher at altitude at 75 W, suggesting hyperventilation occurred at this workload. CO and SV only differed at the two locations during rest. The pregnant women did not experience the typical hyperventilation observed at altitude. The FHR tracings were reactive at both sea level and altitude and the were no significant differences between the two locations. After exercise altitude, one subject had a fetal bradycardia to 105 beats/min for 2 min during which the FHR remained reactive. One subject had uterine activity, which required observation, which resolved without sequela. The ability of pregnant women to perform submaximal exercise at mild or moderate intensity at altitude does not seem to be adversely affected, but it does appear that maximal aerobic capacity is limited in sedentary pregnant women in their third trimester.
Ballew 1984 La Paz, Bolivia	Modified Harvard step test performed at steady-state submaximal level was performed at a clinic in La Paz (3200–4000 m). Step height was 20 cm to compensate for height of the subjects and stage of pregnancy. Subjects stepped at a pace of 76 beats per minute for 6 min. Some results excluded if a subject was unwilling or unable to maintain the stepping pace. Expired air was collected in the last 2 min of the test and HR measured before and after the test. HGB and HCT were measured in the subjects at 8 months. The subjects’ HT, WT, and skinfolds were measured, but timing not indicated. Newborn birth WT, crown-heel length, and skinfolds were measured within 30 h of birth.	Exercise intensity during the stepping test was equivalent to mild to moderate intensity although the range of intensity varied greatly. Subject WT was correlated with exercise test performance and sum of the newborn skinfolds. Ventilation equivalent and maximal HR tended to be negatively correlated with HGB and HCT, but not significantly. When controlled for subject’s WT, $\dot{V}$O_2_, ventilation equivalent and RQ were not related to the newborns’ WT, length, or skinfolds. The maximum HR reached at the end of the exercise test tended to be positively related to the newborn brachial skinfold but did not reach significance. Some limitations of the study were the small sample size, the submaximal exercise test, and that the subjects were native and thus, acclimatized to the high altitude. They hypothesis that women who performed better on the submaximal exercise test at high altitude would have larger babies was not supported. In addition, the evidence did not support the hypothesis that there would be a positive correlation between newborn composition and ventilation equivalent from pregnant individuals experiencing hyperventilation and a resulting increase in arterial saturation because of being at altitude. More research on pregnant individuals who recently migrated or are acutely novel to high altitude is needed.
Davenport, 2018 Everest Base Camp, Nepal	Physical and sleep activity was measured daily with an ActiGraph triaxial accelerometer. Weight, HGB and HCT were measured each morning in the fasting state. RR, HR, pressure of end tidal CO_2_, SpO_2,_ and BP were then measured in a quiet room. The BP was used to calculate pulse pressure and mean arterial pressure. The participant completed the Lake Louise acute mountain sickness questionnaire each morning.	Participant’s BMI was 26.6–27.3 kg/m^2^. The participant performed MVPA ~270 min/day, which was approximately 20–30% of waking hours. A slightly lower level of activity was performed by the participant 10 months later during PP on a subsequent trip. Compared to PP, the participant’s RR was 16–21 breaths/min, indicating hyperventilation. The mean arterial pressure was unchanged during ascent and descent and was between 76 to 86 mmHg. The participant’s HR of 69 to 77 beats/min was lower than lowlander’s typical tachycardia seen in the third trimester and was similar to the subsequent PP values. Unknown if this is particular to Sherpas or because the participant was a trained athlete. Sherpas typically have physiological and pregnancy-induced hemodilution and the participant was no different. The lowest SpO_2_ of 83% was observed after spending the night at 5160 m and after one of the participant’s most active days. The SpO_2_ values were similar prenatally and PP. There was reduced fetal movement at 5300 m but normalized upon the return to 4370 m. Participant reported no mountain sickness. Participant delivered at 42 weeks via emergency caesarean section secondary to nuchal cord and associated fetal distress. Fetal birth weight was 3.2 kg. A year later, the baby was developing normally and did not have any health concerns.
Huch 1996 (Baumann 1985 and Bung, 1987 as reported in Huch) Swiss Mountains and lab, Switzerland	First Study: Participants were driven 130 km from ~408 m (1339 ft) to valley cable car station located at 1080 m (3500 ft). They rode up the cable car in a standing position to 2228 m (7250 ft) in 10 min. After 5 min rest, they rode a bicycle ergometer for 3 min at 25 W, followed by 5 min of rest. The car descended back to the valley and the 3- minute session was repeated. TcPO_2_ and PCO_2_, RR, HR, FHR, and uterine contractions were measured continuously. BP was measured intermittently. Second Study: Participants complete the same protocol at 50 W in a low-pressure chamber simulating altitudes of 1000 m (3500 ft) and 2200 m (7250 ft).	First Study: TcPO_2_ was 71 and 58 mm Hg at the valley and mountain stations, respectively. PCO_2_ was relatively unchanged between the two altitudes or exercise sessions. It did not decrease during exercise ruling out significant hyperventilation. RR was unchanged between the two stations. RR increased from 11 to 20 breaths/min during exercise. The mean resting HR was 101 beats/min. At both stations, mean HR increased from 103 to 128 beats/min during exercise. Systolic BP increased 13 and 27 mm Hg during the valley and mountain exercise sessions, respectively, with diastolic increasing in a similar fashion. Mild uterine contractions were felt by six of the subjects and were unchanged during exercise. The mean FHR increased from 138 to 142 beats/min. during the ascent and was 144 beats/min after exercise. One subject, who smoked at altitude, had a slight fetal bradycardia, and reduced FHR variability after the mountain exercise session, with the FHR returning to normal on the descent. Second Study: Results were similar to the first study except the RR and HRs were slightly higher during exercise. FHR was normal in five of the six pregnant individuals. The sixth pregnant individual, who smoked throughout pregnancy, had a 2 min bradycardia after completing the 2200 m simulated portion of the protocol. Recommendations included lowlanders avoiding altitudes greater than 2500 m for first 4 to 5 days and to exercise at lower altitude levels after initial exposure to altitude.
Niermeyer 1999 Colorado, United States	A Delphi survey was distributed to obstetrical care providers who worked in mountain communities and a Denver referral services inquiring about observed pregnancy complications and risk factors for pregnant individual who visit high altitude.	The rate of acute mountain sickness was the same, if not lower, than the general population. Risk factors for complications for altitude visitors included dehydration and vigorous exercise. The change in left of gravity and changes in body mass may adversely affect balance and coordination leading to increased risk of falling and injury. The respondents recommended a 3 to 4 day acclimatization period before beginning any exercise and short-term visitors should reduce or not exceed their usual level of intensity. Some providers recommended full acclimatization before exercising above 3000 m.
Pivarnik 1992 Utah, United States	A radial artery cannulation was used to obtain arterial blood gases at rest and during exercise. Minute ventilation, alveolar ventilation, dead space ventilation, oxygen uptake, carbon dioxide output, TV, and RR, were also obtained at rest and exercise. After the cannulation was placed and the participants rested, they performed two cycle ergometer (C1 = 50 W; C2 = 75 W) and two treadmill (T1 = 67 m·min^−1;^ 2.5% grade, T2 = 67 m·min^−1;^ 12% grade) tests. A 10 min rest period was provided between each 6 min test. Metabolic and respiratory values were obtained in the fifth and sixth minute of each test. Blood samples were obtained during minute 5. The entire test protocol was repeated at 12 weeks PP. Test took place at 1388 m altitude.	During pregnancy, minute ventilation, alveolar ventilation, TV and ventilatory equivalent for carbon dioxide were greater during exercise compared to PP. Minute ventilation was increased by increases in TV instead of RR. Ventilatory dead space did not differ between exercise and rest or between pregnancy and PP. The increased minute ventilation would not be beneficial to gas exchange unless alveolar ventilation increased, which was observed. The average arterial pH was significantly higher (0.04 units) in pregnancy compared to PP. The arterial pH decreased from rest to exercise during both pregnancy and PP with the greatest decrease occurring during the 75 W cycling test. It appears that pregnancy status did not influence the pH changes during exercise. PaCO_2_ was significantly lower during pregnancy. However, it was unchanged from rest to exercise, unlike PP for which PaCO_2_ decreased. Smaller decreases in arterial bicarbonate were observed during pregnancy compared to PP during exercise. The researchers concluded that the acid–base response to cycling and treadmill walking were not compromised in late pregnancy and gestational hyperventilation helped maintain PaCO_2_ and arterial bicarbonate during pregnancy.

Note. BMI = body mass index; BP = blood pressure; CO = cardiac output; ECG = electrocardiogram; FHR = fetal heart rate; HCT = hematocrit; HGB = hemoglobin; HR = heart rate; HT = height; MVPA = moderate and vigorous physical activity; PaCO_2_ = arterial carbon dioxide tension or partial pressure of arterial carbon dioxide; PCO_2_ = carbon dioxide tension or partial pressure of carbon dioxide PP = postpartum: RQ = respiratory quotient; RR = respiratory rate (ventilatory frequency); SpO_2 =_ peripheral oxyhemoglobin saturation; SV = stroke volume; TcPO_2_ *=* Transcutaneous oxygen tension; TV = tidal volume; $\dot{V}$CO_2_ = elimination of carbon dioxide; $\dot{V}$O_2_ = oxygen consumption; WT = weight.

## Appendix B

**Table A3:** JBI Critical Appraisal Checklist for quasi-experimental studies—Artal et al. (1995) study. Citation: Artal, R.; Fortunato, V.; Welton, A.; Constantino, N.; Khodiguian, N.; Villalobos, L.; Wiswell, R. A Comparison of Cardiopulmonary Adaptations to Exercise in Pregnancy at Sea Level and Altitude. *Am. J. Obstet. Gynecol.* **1995**, *172*, 1170–1178; discussion 1178–1180, doi:10.1016/0002-9378(95)91475-7.

	Yes	No	Unclear	Not Applicable
Is it clear in the study what is the ‘cause’ and what is the ‘effect’ (i.e., there is no confusion about which variable comes first)?	x	□	□	□
2. Were the participants included in any comparisons similar?	□	x	□	□
3. Were the participants included in any comparisons receiving similar treatment/care, other than the exposure or intervention of interest?	□	x	□	□
4. Was there a control group?	□	x	□	□
5. Were there multiple measurements of the outcome both pre and post the intervention/exposure?	□	x	□	□
6. Was follow up complete and if not, were differences between groups in terms of their follow up adequately described and analyzed?	□	x	□	□
7. Were the outcomes of participants included in any comparisons measured in the same way?	x	□	□	□
8. Were outcomes measured in a reliable way?	x	□	□	□
9. Was appropriate statistical analysis used?	x	□	□	□
Overall appraisal: Include x Exclude □ Seek further info □.				

Comments (Including reason for exclusion). Study was included because it was one of the few available and guidelines were based on this study. Recruitment of 10 subjects was not described and data on peak exercise variables were obtained on only 7 subjects. Information concerning if the three subjects were different from the other 10 subjects was not provided. There was no counterbalancing in this study. © JBI, 2020. All rights reserved. JBI grants use of these tools for research purposes only. All other enquiries should be sent to jbisynthesis@adelaide.edu.au.

**Table A4:** JBI Critical Appraisal Checklist for quasi-experimental studies—Ballew (1984) study. Citation: Ballew, C.C. The Effect of High Altitude Hypoxia on Newborn Body Composition in Two Populations in Bolivia. Ph.D. Thesis, The Pennsylvania State University, State College, PA, USA, 1984.

	Yes	No	Unclear	Not Applicable
Is it clear in the study what is the ‘cause’ and what is the ‘effect’ (i.e., there is no confusion about which variable comes first)?	x	□	□	□
2. Were the participants included in any comparisons similar?	□	□	□	x
3. Were the participants included in any comparisons receiving similar treatment/care, other than the exposure or intervention of interest?	□	x	□	□
4. Was there a control group?	□	x	□	□
5. Were there multiple measurements of the outcome both pre and post the intervention/exposure?	□	x	□	□
6. Was follow up complete and if not, were differences between groups in terms of their follow up adequately described and analyzed?	□	□	x	□
7. Were the outcomes of participants included in any comparisons measured in the same way?	x	□	□	□
8. Were outcomes measured in a reliable way?	□	x	□	□
9. Was appropriate statistical analysis used?	x	□	□	□
Overall appraisal: Include X Exclude □ Seek further info □.				

Comments (Including reason for exclusion). Study was included because it is one of the few in which individuals who are pregnant were tested during exercise at altitude. The researcher was vague about the number of participants who were unwilling to unable to keep up the pace or complete the entire exercise test. Information about the validation of the step test, which was modified by decreasing the step height, was not provided. The participants were compared to participants from another part of the study, but those results were not relevant to this review. © JBI, 2020. All rights reserved. JBI grants use of these tools for research purposes only. All other enquiries should be sent to jbisynthesis@adelaide.edu.au.

**Table A5:** JBI Critical Appraisal Checklist for case reports—Davenport et al. (2018). Citation: Davenport, M.H.; Steinback, C.D.; Borle, K.J.; Matenchuk, B.A.; Vanden Berg, E.R.; de Freitas, E.M.; Linares, A.M.; O’Halloran, K.D.; Sherpa, M.T.; Day, T.A. Extreme Pregnancy: Maternal Physical Activity at Everest Base Camp. *J. Appl. Physiol.* **2018**, *125*, 580.

	Yes	No	Unclear	Not Applicable
Were patient’s demographic characteristics clearly described?	x	□	□	□
2. Was the patient’s history clearly described and presented as a timeline?	x	□	□	□
3. Was the current clinical condition of the patient on presentation clearly described?	x	□	□	□
4. Were diagnostic tests or assessment methods and the results clearly described?	x	□	□	□
5. Was the intervention(s) or treatment procedure(s) clearly described?	x	□	□	□
6. Was the post-intervention clinical condition clearly described?	x	□	□	□
7. Were adverse events (harms) or unanticipated events identified and described?	x	□	□	□
8. Does the case report provide takeaway lessons? Overall appraisal: Include X Exclude □ Seek further info □.	x	□	□	□

Comments (Including reason for exclusion). This is one of the few studies conducted on strenuous activity at altitude. This study was conducted at 3440 to 5160 m which is much higher than most elite athletes train. © JBI, 2020. All rights reserved. JBI grants use of these tools for research purposes only. All other enquiries should be sent to jbisynthesis@adelaide.edu.au.

**Table A6:** JBI Critical Appraisal Checklist for quasi-experimental studies—Huch (1996) study #1. Citation: Huch, R. Physical Activity at Altitude in Pregnancy. *Semin. Perinatol.* **1996**, *20*, 303–314, doi:10.1016/S0146-0005(96)80023-1.

	Yes	No	Unclear	Not Applicable
Is it clear in the study what is the ‘cause’ and what is the ‘effect’ (i.e., there is no confusion about which variable comes first)?	x	□	□	□
2. Were the participants included in any comparisons similar?	□	□	□	x
3. Were the participants included in any comparisons receiving similar treatment/care, other than the exposure or intervention of interest?	□	x	□	□
4. Was there a control group?	□	x	□	□
5. Were there multiple measurements of the outcome both pre and post the intervention/exposure?	□	x	□	□
6. Was follow up complete and if not, were differences between groups in terms of their follow up adequately described and analyzed?	x	□	□	□
7. Were the outcomes of participants included in any comparisons measured in the same way?	x	□	□	□
8. Were outcomes measured in a reliable way?	□	□	x	□
9. Was appropriate statistical analysis used? Overall appraisal: Include X Exclude □ Seek further info □.	□	□	x	□

Comments (Including reason for exclusion). Study was included because it was one of the few available and guidelines were based on this study. This study was conducted and reported originally in another language. Huch reported the study in English and did not provide detailed methodology. Twelve individuals participated in the study and recruitment information was not provided. Bicycle tests were not counter-balanced. The transcutaneous electrode used may not have been appropriate for detecting a change during the short test. The exercise tests lasted only 3 min. Not all of the data were provided. © JBI, 2020. All rights reserved. JBI grants use of these tools for research purposes only. All other enquiries should be sent to jbisynthesis@adelaide.edu.au.

**Table A7:** JBI Critical Appraisal Checklist for quasi-experimental studies—Huch (1996) study #2. Citation: Huch, R. Physical Activity at Altitude in Pregnancy. *Semin. Perinatol.* **1996**, *20*, 303–314, doi:10.1016/S0146-0005(96)80023-1. STUDY #2.

	Yes	No	Unclear	Not Applicable
Is it clear in the study what is the ‘cause’ and what is the ‘effect’ (i.e., there is no confusion about which variable comes first)?	x	□	□	□
2. Were the participants included in any comparisons similar?	□	□	□	x
3. Were the participants included in any comparisons receiving similar treatment/care, other than the exposure or intervention of interest?	□	x	□	□
4. Was there a control group?	□	x	□	□
5. Were there multiple measurements of the outcome both pre and post the intervention/exposure?	□	x	□	□
6. Was follow up complete and if not, were differences between groups in terms of their follow up adequately described and analyzed?	x	□	□	□
7. Were the outcomes of participants included in any comparisons measured in the same way?	x	□	□	□
8. Were outcomes measured in a reliable way?	□	□	x	□
9. Was appropriate statistical analysis used? Overall appraisal: Include x Exclude □ Seek further info □.	□	□	x	□

Comments (Including reason for exclusion). Study was included because it was one of the few available and guidelines were based on this study. This study was conducted and reported originally in another language. Huch reported the study in English and did not provide detailed methodology. Similar methodology that was used in Study 1 was used in Study 2, except the testing was done in a low-pressure chamber. Detailed methodology and data results were not provided. © JBI, 2020. All rights reserved. JBI grants use of these tools for research purposes only. All other enquiries should be sent to jbisynthesis@adelaide.edu.au.

**Table A8:** Rapid Critical Appraisal Questions for descriptive studies—Neirmeyer (1999) study. **Citation:** Niermeyer, S. The Pregnant Altitude Visitor. *Adv. Exp. Med. Biol.* **1999**, *474*, 65–77, doi:10.1007/978-1-4615-4711-2_5.

**Validity**
**Are the results of the study valid?**
Were study/survey methods appropriate for the question? Yes No Unknown Was sampling methods appropriate for the research question? Yes No Unknown Were sample size implications on study results discussed? Yes No Unknown Were variables studied appropriate for the question? Yes No Unknown o Dependent variables are: *Observed pregnancy complications, risk factors for individuals who are pregnant who visit high altitude.* o Independent variables are: *None* Were outcomes appropriate for the question? Yes No Unknown Were valid and reliable instruments used to measure outcomes? Yes No Unknown Were the chosen measures appropriate for study outcomes? Yes No Unknown Were outcomes clearly described? Yes No Unknown Did investigators and/or funding agencies declare freedom from conflict of interest? Yes No Unknown
**Reliability** **What are the results?**
What were the main results of the study? o Was there statistical significance? Explain. No, it was a Delphi survey. o Was there clinical significance? Explain. Yes, risk factors and pregnancy complications were identified. Were safety concerns, including adverse events and risk/benefit described? Yes No Unknown
**Applicability**
**Will the results help me in caring for my patients?**
Are the results applicable to my patient population? Yes No Unknown Will my patients’ and families’ values and beliefs be supported by the knowledge gained from the study? Yes No Unknown
**Would you use the study results in your practice to make a difference in patient outcomes?**
If yes, how? *Yes, to advise patients of possible complications and what symptoms to look for.* If yes, why? *By advising patients of problems of traveling to altitude, the provider can make recommendations for self-care to avoid developing a pregnancy-related complication.* If no, why not?
**Additional Comments/Reflections:** The results from a Delphi survey of 12 obstetrical providers were provided in a narrative literature review. Methodology for the survey was not reported. This study discussed reports of performing exercise at altitude and the possible complications observed in the past.
**Recommendations for article use within a body of evidence:** © Fineout-Overholt and Gallagher-Ford, 2012. This form may be used for educational, practice change and research purposes without permission.

**Table A9:** JBI Critical Appraisal Checklist for quasi-experimental studies—Pivarnik et al. (1992) study. Citation: Pivarnik, J.M.; Lee, W.; Spillman, T.; Clark, S.L.; Cotton, D.B.; Miller, J.F. Maternal Respiration and Blood Gases during Aerobic Exercise Performed at Moderate Altitude. *Med. Sci. Sports Exerc.* **1992**, *24*, 868–872.

	Yes	No	Unclear	Not Applicable
Is it clear in the study what is the ‘cause’ and what is the ‘effect’ (i.e., there is no confusion about which variable comes first)?	x	□	□	□
2. Were the participants included in any comparisons similar?	x	□	□	□
3. Were the participants included in any comparisons receiving similar treatment/care, other than the exposure or intervention of interest?	□	x	□	□
4. Was there a control group?	□	x	□	□
5. Were there multiple measurements of the outcome both pre and post the intervention/exposure?	x	□	□	□
6. Was follow up complete and if not, were differences between groups in terms of their follow up adequately described and analyzed?	x	□	□	□
7. Were the outcomes of participants included in any comparisons measured in the same way?	x	□	□	□
8. Were outcomes measured in a reliable way?	x	□	□	□
9. Was appropriate statistical analysis used? Overall appraisal: Include x Exclude □ Seek further info □.	x	□	□	□

Comments (Including reason for exclusion). Study was included because it was one of the few available that looked at exercise at altitude prenatally and post-partum. Seven subjects from a different arm of the study were recruited. It was not described why these seven were chosen. The testing was conducted at 1388, which is consider low altitude. This study does provide prenatal and postpartum results. Counterbalancing in this study was not possible. © JBI, 2020. All rights reserved. JBI grants use of these tools for research purposes only. All other enquiries should be sent to jbisynthesis@adelaide.edu.au.
